# Case Report: Concurrent *de novo* pathogenic variants in the *LMNA* gene as a cause of sporadic partial lipodystrophy

**DOI:** 10.3389/fgene.2024.1468878

**Published:** 2024-11-28

**Authors:** José L. Santos, José Patricio Miranda, Carlos F. Lagos, Víctor A. Cortés

**Affiliations:** ^1^ Department of Nutrition, Diabetes and Metabolism, School of Medicine, Pontificia Universidad Católica de Chile, Santiago, Chile; ^2^ Department of Health Sciences, Institute for Sustainability and Food Chain Innovation (IS-FOOD), Public University of Navarre, Pamplona, Spain; ^3^ Bupa Lab, Part of Bupa Chile, Santiago, Chile; ^4^ Chemical Biology and Drug Discovery Laboratory, Escuela de Química y Farmacia, Facultad de Medicina y Ciencia, Universidad San Sebastián, Santiago, Chile; ^5^ Centro Ciencia and Vida, Fundación Ciencia and Vida, Santiago, Chile

**Keywords:** lipodystrophy, LMNA, *de novo*, exome, pathogenic mutation

## Abstract

**Introduction:**

Inherited lipodystrophies are a group of rare diseases defined by severe reduction in adipose tissue mass and classified as generalized or partial. We report a non-familial (sporadic) case of partial lipodystrophy caused by a novel genetic mechanism involving closely linked *de novo* pathogenic variants in the *LMNA* gene.

**Methods:**

A female adult with partial lipodystrophy and her parents were evaluated for gene variants across the exome under different mendelian inheritance models (autosomal dominant, recessive, compound heterozygous, and X-linked) to find pathogenic variants. Body composition was assessed via dual-energy X-ray absorptiometry (DXA).

**Results:**

The patient showed absence of adipose tissue in the limbs; preservation of adiposity in the face, neck, and trunk; muscular hypertrophy, hypertriglyceridemia and insulin resistance. DXA revealed a fat mass of 15.4%, with android-to-gynoid ratio, trunk/limb, and trunk/leg ratios exceeding the published upper limits of 90% reference intervals. Two heterozygous missense *de novo* pathogenic variants *in cis* within the *LMNA* gene were found in the proband: p.Y481H and p.K486N (NP_733821.1). These variants have functional effects and were reported in inherited Emery-Dreifuss muscular dystrophy 2 (p.Y481H) and familial partial lipodystrophy type 2 (p.K486N). Molecular modeling analyses provided additional insights into the protein instability conferred by these variants in the lamin A/C Ig-like domain.

**Conclusion:**

In a case of sporadic partial lipodystrophy, we describe two concurrent *de novo* pathogenic variants within the same gene (*LMNA*) as a novel pathogenic mechanism. This finding expands the genetic and phenotypic spectrum of partial lipodystrophy and laminopathy syndromes.

## Introduction

Nucleophilic A-type lamins A and C, usually referred to as lamin A/C, are integral components of the nuclear lamina, a multiprotein filamentous structure associated with the inner nuclear membrane. Because of this crucial structural role, lamins A/C are expressed in virtually all mammalian cells, being expressed in equal quantities in the nuclear lamina. Lamins A and C are encoded by the *LMNA* gene (12 exons on chromosome 1q22; transcript NM_170,707) and are generated by alternative splicing of exon 10. Prelamin A is extensively processed and cleaved at C-terminal residues by *ZMPSTE24*, leading to the mature lamin A protein (Dittmer and Misteli, 2011; Genschel and Schmidt, 2000; Casasola et al., 2016). Thus, lamin A (canonical sequence of 664 amino acids; NP_001393912.1; UniProtKB: P02545-1; https://www.uniprot.org/uniprot/P02545) and lamin C (572 amino acids; NP_001393913.1; UniProtKB: P02545-2) share their first 566 N-terminal amino acids, with lamin C having differences in sequence for residues 567–572 and lacking the residues 573–664 in comparison to lamin A.

Pathogenic gene variants in the *LMNA* gene cause a group of heterogeneous human diseases collectively termed laminopathies (Crasto et al., 2020). These disorders include cardiac diseases, Emery-Dreifuss muscular dystrophies, limb-girdle muscular dystrophy (renamed as Emery-Dreifuss muscular dystrophy 2; EDMD2, OMIM: #181350) (Straub et al., 2018), Charcot-Marie-Tooth type 2, progeroid syndromes, and familial partial lipodystrophies (Genschel and Schmidt, 2000; Nishiuchi et al., 2017; Florwick et al., 2017). In total, more than 600 *LMNA* disease-causing gene variants have been reported (https://www.hgmd.cf.ac.uk/), resulting in at least 15 different human pathological phenotypes (Crasto et al., 2020; Fernandez-Pombo et al., 2023a). Although the mechanisms by which *LMNA* variants result in such multiple phenotypes remain unknown, it is increasingly recognized that lamins A/C have biological functions exceeding mere structural roles in the nuclear lamina, including involvement in gene transcription regulation, chromatin organization, and DNA replication (Crasto et al., 2020).

Lipodystrophies are a group of rare diseases characterized by severe reduction and redistribution of adipose tissue. Depending on the extent of adipose paucity, lipodystrophies can be generalized or partial, and depending on their etiology, they have been classically classified as congenital, familial, or acquired (Brown et al., 2016; Cortés et al., 2014; Cortés and Santos, 2019). Congenital generalized lipodystrophy (CGL) is the most extreme form of human genetic leanness and is caused by homozygous pathogenic variants in the *AGPAT2*, *BSCL2*, *CAV1*, or *PTRF* (*CAVIN1*) genes (Cortés et al., 2014; Cortés et al., 2009; Santos and Cortés, 2021). Patients with CGL develop severe insulin resistance, diabetes, hepatic steatosis, hypertriglyceridemia, and hyperphagia (Akinci et al., 2018). On the other hand, familial partial lipodystrophy (FPLD) is characterized by loss of fat restricted to some anatomic regions, typically the extremities, with preservation or even enlargement of the remaining adipose depots, typically in the abdomen. Patients with FPLD also develop insulin resistance, diabetes mellitus, fatty liver, and dyslipidemia, but usually to a lesser severity than those with CGL (Vasandani et al., 2022) without exhibiting hyperphagia (Monteiro et al., 2017). FPLD has been linked to pathogenic variants in the *LMNA*, *CIDEC*, *LIPE*, *PCYT1A*, *PPARG*, *AKT2*, *POLD1*, and *KCNJ6* genes (Brown et al., 2016). The commonest mode of inheritance of FPLD is autosomal dominant, with very rare cases of homozygous disease-causing variants reported in specific populations (Fernandez-Pombo et al., 2023a). The more frequent FPLD subtype is caused by heterozygous *LMNA* pathogenic variants, termed FPLD type 2 (FPLD2) or Dunnigan variety (Vasandani et al., 2022). This lipodystrophy is characterized by the absence of adipose tissue in the limbs, muscle hypertrophy and preserved or increased adiposity in the face, neck, and trunk. FPLD has been reported to be ∼3–4 times more frequent in women than in men (Akinci et al., 2018; Fernandez-Pombo et al., 2023b).

Herein, we describe a female patient with partial lipodystrophy, with adipose depletion in the extremities but preservation in the face/trunk, hypertriglyceridemia, and severe insulin resistance. Exome sequencing revealed two heterozygous missense *de novo* pathogenic variants *in cis* within the *LMNA* gene: p.Y481H and p.K486N. These rare single nucleotide variants have been previously associated with dominant limb-girdle muscular dystrophy (Emery-Dreifuss muscular dystrophy 2) (Brown et al., 2016; Kitaguchi et al., 2001) and partial lipodystrophy (Shackleton et al., 2000), and reported to affect lamin A/C function by *in vitro* studies (Anderson et al., 2021; Simon et al., 2013). Using molecular modeling, molecular dynamics simulation, and predictive bioinformatic analysis, we propose mechanisms by which these variants lead to lamin A/C Ig-like domain instability and affect lamin A/C function. This is the first report of a sporadic partial lipodystrophy syndrome of genetic origin caused by concurrent *de novo* pathogenic variants within the same gene.

## Subjects and methods

This research is based on a case-parent trio ascertained via the proband, who is a 24-year-old female patient referred to our center because of extreme leanness, hyperinsulinemia, oligomenorrhea, and hypertriglyceridemia (Table 1). The patient was born to nonconsanguineous parents and works as an administrative assistant. She was concerned about their general health status and future implications but felt that her current clinical status did not interfere with her daily life. Physical exploration revealed a notorious deficiency of subcutaneous adipose tissue in both the upper and lower extremities, with preservation of truncal and facial adiposity. She presented intense acanthosis nigricans at the base of the neck and axillar folds, hirsutism, and diffuse acneiform skin lesions. Despite the patient’s regional lipoatrophy, body weight remained within the normal range. Both parents showed normal adiposity and did not present metabolic abnormalities. The patient had no family history of low body weight, diabetes, dyslipidemia, or cardiovascular or muscle disease. Because of oligomenorrhea, she was prescribed oral contraceptives (ethynyl estradiol 0.035 mg, cyproterone 2 mg). Clinical assessment revealed no muscle pain or weakness.

**TABLE 1:** Clinical features of the patient with sporadic partial lipodystrophy of genetic origin.

Patient		
Anthropometry		
Weight	52 kg	
Stature	156 cm	
Body mass index	21.4 kg/m^2^	
Body composition (DXA)		
Body fat mass	8 kg	
Body fat %	15.4%	
Fat mass index	3.3 kg/m^2^	
		90% Reference intervals^a^
Android-to-gynoid (A/G) ratio	0.59	0.20–0.47
Fat Trunk/limb ratio	1.79	0.71–1.21
Fat Trunk/leg ratio	1.38	0.72–1.10
Laboratory biochemistry tests in plasma		Reference values^b^
Total cholesterol	240 mg/dL	<200 mg/dL
Triglycerides	484 mg/dL	<150 mg/dL
HDL cholesterol	38 mg/dL	>60
Fasting glucose	93 mg/dL	<100 mg/dL
Post-load glucose (2 h, 75 g glucose)	119 mg/dL	<140 mg/dL
Fasting insulin	50 μU/mL	2.6–24.9 μU/mL
Post-load insulin (2 h, 75 g glucose)	580 μU/mL	-
HbA1c	5.3%	<5.7%
Creatinine	0.7 mg/dL	0.5–0.9 mg/dL

^a^ 90% central reference intervals (10th - 90th percentiles) for women aged >20–29 years (21).

^b^ Desirable reference values and ranges as reported by Laboratorio Clínico UC-Christus (Santiago, Chile) (https://appsinfex.ucchristus.cl/Sinfex/#/list).

Body composition with dual-energy X-ray absorptiometry (DXA; GE-Healthcare Lunar DPX-NT scanner) revealed a fat mass of 15.4%. Android-to-gynoid (A/G) ratio (android fat mass/gynoid fat mass), trunk/limb, and trunk/leg ratios exceeded the upper limits of 90% reference intervals using the same DXA equipment (Imboden et al., 2017), indicating a fat distribution pattern characterized by central adiposity and excessive accumulation of fat in the trunk relative to the arms and legs. The bone mineral density was 1.08 g/cm^2^ (z-score = 0). Abdominal ultrasonography revealed splenomegaly and diffuse liver enlargement associated with mild steatosis. Liver function tests were within the normal range, including transaminases, total bilirubin, and alkaline phosphatase levels.

Massively parallel sequencing (MPS) was used to interrogate gene variants in the proband´s exome from DNA isolated from blood leukocytes. We used SureSelect XT v6 (Agilent Technologies) for exome capture. Libraries were sequenced on the Illumina HiSeq equipment in Theragen-Etex (http://www.theragenetex.net/) to a raw average sequencing depth of >100X and an exome coverage of 10X >98%. Each of the exomes of the trio resulted in >76 million reads with >96% of the Q20 quality score (read length = 101 × 2). Alignments were made using the hg19 human genome assembly (GRCh37). Sequence primary data analysis, including read alignment and variant calling, was also performed by Theragen-Etex. Mutations were confirmed via Sanger sequencing.

We used publicly available bioinformatic tools (Alphamissense, SIFT, Polyphen-2, CADD, REVEL, MetaLR, and MutationAssessor) to predict the effect of missense variants p.Y481H and p.K486N on protein function (available at https://alphamissense.hegelab.org/ and https://www.ensembl.org/). Homology analysis was carried out with WebLogo (https://weblogo.berkeley.edu/), PhyloP (https://genome.ucsc.edu/cgi-bin/hgTrackUi?db=hg19&g=cons100way), CONSURF (https://consurf.tau.ac.il/), and EVE (https://evemodel.org/proteins/LMNA_HUMAN) programs (Lindquist et al., 2022). The supplementary material shows extended methods for the molecular modeling and dynamics simulation of Lamin A/C mutants.

## Results

### Exome sequencing and genetic analysis

**FIGURE 1:**
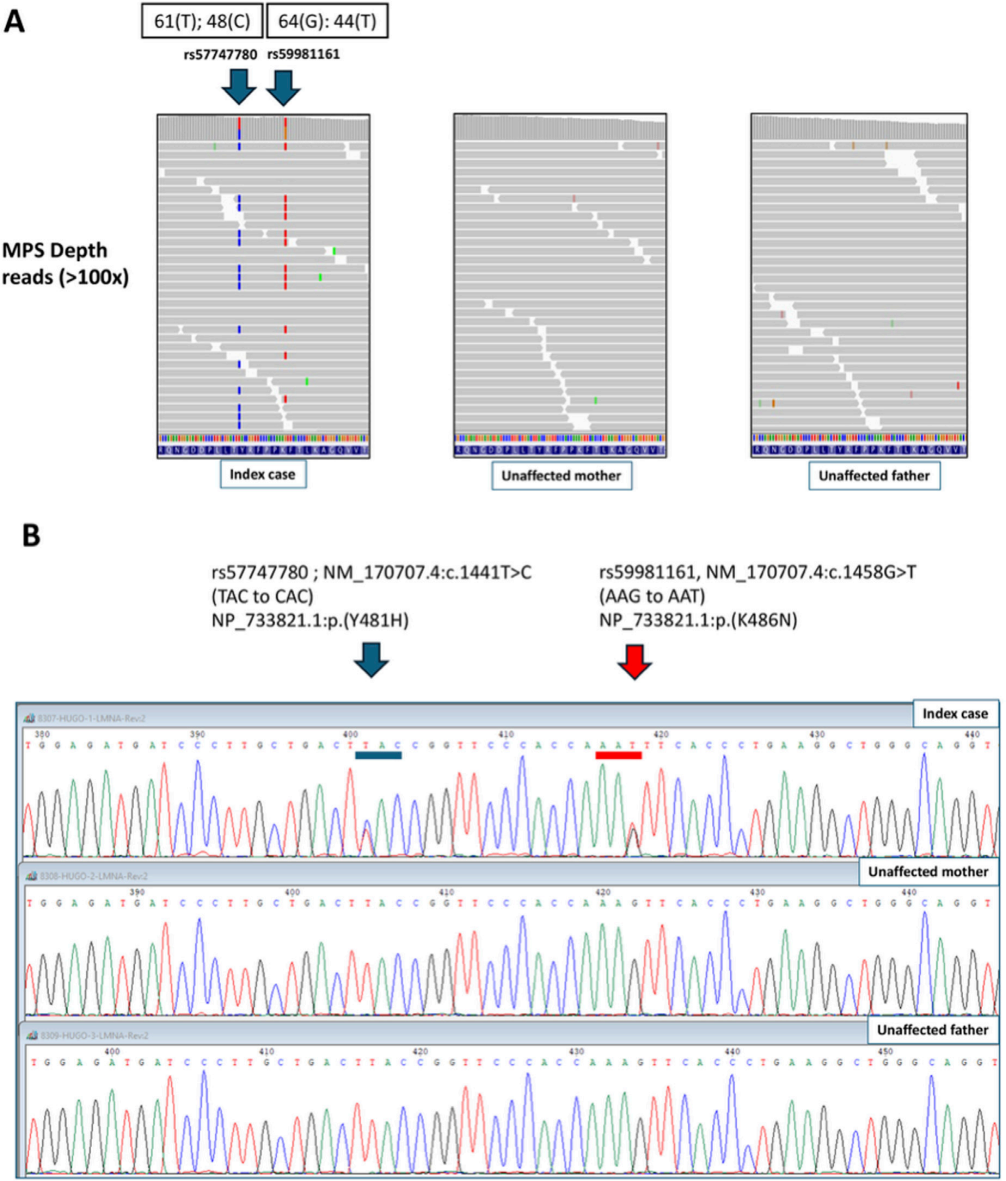
Identification of two *de novo* missense pathogenic variants (p.Y481H and p.K486N) in the *LMNA* gene of the proband with partial lipodystrophy. **(A)** Massively Parallel Sequencing (MPS) of the proband and parents. **(B)** Sanger sequencing of the proband and parents.

Extensive exome sequence analysis of the patient and her parents using different Mendelian inheritance models (autosomal dominant, recessive, compound heterozygous, and X-linked) revealed no inherited pathogenic variants linked to the patient’s phenotype. However, we found two missense rare single nucleotide variants in heterozygosity in the *LMNA* gene only in the proband, corresponding, therefore, to *de novo* variants (NCBI Gene ID: 4000; HGNC ID: 6636; Ensembl Gene ID: ENSG00000160789). At the DNA level, the two variants found in this study are NM_170,707.4:c.1441T>C (rs57747780) and NM_170707.4:c.1458G>T (rs59981161). In terms of amino acid changes the two variants are NP_733821.1:p. (Tyr481His) (also named p.Y481H) and NP_733821.1:p. (Lys486Asn) (also named p.K486N) (Figure 1). Genetic variants in the *LMNA* gene are reported following international recommendations (https://varnomen.hgvs.org/). One-letter coding for amino acids was preferentially used for comparison with the other studies.

The clinical significance of the variant p.Y481H is not provided by ClinVar (https://www.ncbi.nlm.nih.gov/clinvar), while p.K486N is reported to be pathogenic and linked to FPLD2 (Dunnigan type). There is no mention of the population frequencies of these variants, indicating that they correspond to very rare variants. In gnomAD (https://gnomad.broadinstitute.org/), variant rs57747780 was not reported, while rs59981161 was only found in one allele among 1614224 alleles (no homozygotes; allele frequency = 6.19e-7). Based on guidelines on the clinical interpretation of genetic variants (Richards et al., 2015) (https://clinicalgenome.org/working-groups/sequence-variant-interpretation/) and considering all the available information, the online calculator INTERVAR reassigned both variants as “pathogenic” (https://wintervar.wglab.org/).

The depth of the MPS reads in the patient was above 100x for both variants and flanking regions (Figure 1A), providing acceptable reliability to the sequence. The heterozygote status of the patient was confirmed by standard Sanger sequencing using appropriate primers (see supplementary material). Parents did not show these genetic variations (MPS and Sanger sequencing). Additionally, both variants in the patient were always found in the DNA simultaneously in the same reads of the patient (Figure 1A), indicating that they occurred *in cis* (i.e., on the same chromosome). It was not possible to assign the maternal/paternal origin of the haplotype phase, given the short reads of the NGS technique and the lack of informative variants in the vicinity of the pathogenic variants. True paternity in the trio was confirmed with the exome data using a panel of 22 synonymous single nucleotide polymorphisms (SNPs) designed to track samples’ origin and paternity identification (Huang et al., 2024). Such SNPs showed high depth and quality, yielding a combined paternity index of 14,498 and a probability of paternity W = 0.999,931 in the case-parent trio. Population frequencies of SNPs involved in CPI and W calculations were retrieved from genotypes of the MEGA-Illumina array (www.illumina.com) in the Chilean study “Growth and Obesity cohort study” (Miranda et al., 2024). See Supplement Tables S1, S2 for features of SNPs used in paternity analysis and calculation of parentage statistics.

In mendelian inheritance models, true heterozygous inherited variants must fit a theoretical allelic proportion of 50% (percentage of variant reads from the total number of sequencing reads). In the present case, these percentages were 44% for c.1441T > C (p.Y481H) and 41% for c.1458G > T (p.K486N) (Figure 1A), which are slightly lower than the expected 50%. However, it has been reported that such a percentage may vary from 35% to 65% (mean ±2 standard deviations) of true inherited heterozygous variants, as estimated from whole-genome sequencing (NGS) techniques (Acuna-Hidalgo et al., 2015). An exact binomial test to evaluate the null hypothesis of an expected proportion of 50% was carried out using the command *bitesti* in STATA 17.0). This test yielded a *p*-value of 0.25 (rs57747780; p.Tyr481His) and 0.07 (rs59981161; p.Lys486Asn). Then, there is no statistical evidence to discard the hypothesis of true heterozygous status (50% proportion) in the affected case. Taken together, our exome sequencing results (Figure 1A) combined with the typical pattern of heterozygosity shown by Sanger sequencing (Figure 1B) led us to propose that the variants present in this patient originated in a germline *de novo* mutation in the *LMNA* gene in a single chromosome, with no evidence of mosaicism.

### Bioinformatic prediction and homology analysis of lamin A/C variants

Table 2 shows the bioinformatic analysis of the functional effects of p.Y481H and p.K486N according to different prediction algorithms supporting the prediction of deleterious functional effects of both variants, especially for the p.Y481H gene variant. Homology analysis showed high conservation in the amino acid and DNA sequences around the reported mutations across different species (Kitaguchi et al., 2001; Shackleton et al., 2000). We generated a logo plot to visualize the DNA and amino acid sequence conservation using *LMNA* orthologue alignments of 245 species retrieved from the Ensembl genome browse 112 (Supplement Figure S1). The 100-vertebrates’ basewise conservation score was calculated by PhyloP, yielding a score of 7.62 for rs57747780 and 2.26 for rs59981161. A positive PhyloP score indicates evolutionary conservation, with the absolute values score representing the -log *p*-values under a null hypothesis of neutral evolution (Supplement Figure S2). Additionally, Bayesian modeling yielded conservation scores with the CONSURF software: the amino acid conservation score for LMNA_481Y was −0.39 (95%CI: −0.661, −0.149) while the score of 486K was - 0.373 (95%CI: −0.661, −0.250), both corresponding to a grade six of conservation in a scale from 1 (less conserved) to 9 (most conserved) (Supplement Figure S3). Finally, we also used a pathogenicity predictor based on evolutionary variation across species (EVE score of 0, the most benign; 1: the most pathogenic), yielding a score of 0.642 for Y481H (classified as pathogenic) and a score of 0.503 for K486N (classified as uncertain) (Lindquist et al., 2022; Frazer et al., 2021).

**TABLE 2:** Scores of functional effect prediction of p.Y481H and p.K486N mutations in the *LMNA* gene.

	*LMNA* p.Y481H	*LMNA* p.K486N
Alphamissense	0.985	0.986
SIFT	0	0
Polyphen-2	1	0.77
CADD	32	24
REVEL	0.982	0.639
MetaLR	0.979	0.893
MutationAssessor	0.938	0.433

For Alphamissense, the score indicates the predicted probability of the variant being pathogenic (both classified as “likely pathogenic”). In SIFT, a score <0.05 indicated “deleterious” (range: 0–1). For Polyphen-2, a score >0.95 indicated “probably damaging”, while scores from 0.45 to 0.9 indicated “possibly damaging” (range: 0–1). For CADD, variants with scores >30 are predicted to be the 0.1% most deleterious substitutions in the human genome, while a score >20 indicates the 1% most deleterious. For REVEL, scores >0.5 were classified as “likely disease-causing”. For MetaLR and MutationAssessor, scores near 1 were considered “damaging” or “deleterious” (range: 0–1). Predictions based on proteins from the ENST00000368300.9 transcript. For more information, see http://genetics.bwh.harvard.edu/pph2/, https://alphamissense.hegelab.org/ and https://www.ensembl.org/.

### Molecular modeling and dynamics simulation of lamin A/C mutants

**FIGURE 2:**
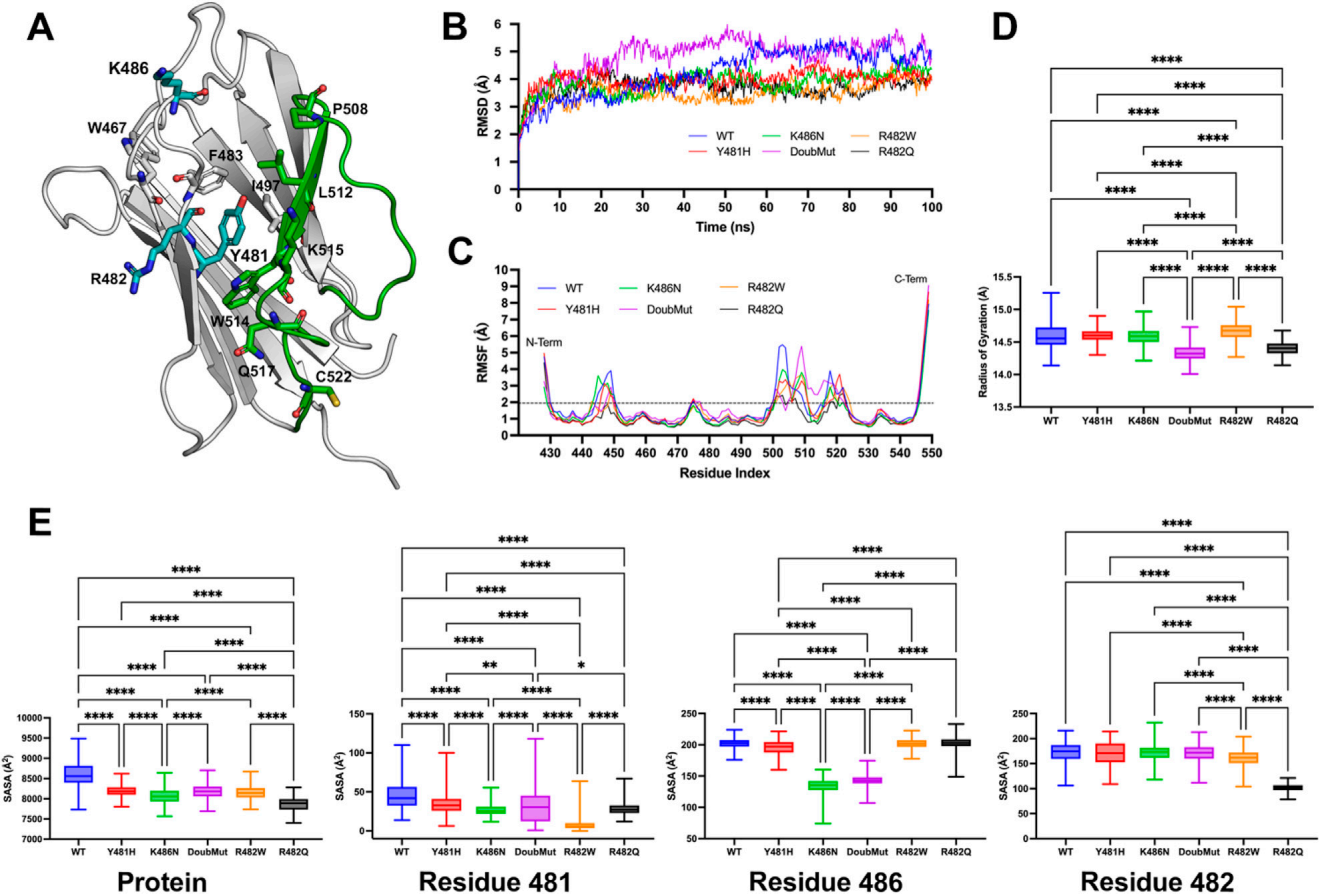
Molecular dynamics simulations of lamin A/C variants **(A)** Structural mapping of the p.Y481H and p.K486N mutations and other relevant residues on the crystal structure of wild-type (WT) lamin A/C protein (PDB ID: 1IVT). The 500–520 loop is shown in green. **(B)** Comparative root-mean-square deviation (RMSD) throughout the simulation for the α-carbon backbone atoms of the wild-type and mutated lamin A/C variants, indicating changes in protein stability. **(C)** Root-mean-square fluctuation (RMSF) analysis of the α-carbon atoms, reflecting the localized flexibility within the wild-type and mutant lamin A/C structures. **(D)** Radius of gyration (RoG) profiles for both the wild-type and mutant lamin A/C variants, showing the compactness of the protein structures during the simulation. **(E)** Solvent accessible surface area (SASA) measurements for wild-type and mutated lamin A/C, including a detailed comparison at the residue level, to assess changes in surface properties due to mutation. The box plots depict the minimum and maximum values (whiskers), the upper and lower quartiles, and the median. The length of the box represents the interquartile range. One-way ANOVA followed by Bonferroni’s multiple comparisons test was performed using GraphPad Prism version 10.3.1 (GraphPad Software Inc.). A *p*-value less than 0.05 was considered to indicate statistical significance (n = 500 frames corresponding to the last 50 ns of the production simulation).

The Ig-like domain of human lamin A/C (PDB id 1IVT, residues 428–549) was used as a starting point for the molecular modeling the p.Y481H and p.K486N variants, as well as for the more frequent pathogenic variants p.R482Q and p.R482W (see supplementary material for methods on molecular modeling and dynamic simulations). Analysis of wild-type and mutated lamins A/C showed that the wild-type Y481 and K486 residues are close within the domain’s three-dimensional conformation, with K486 being highly solvent-exposed and Y481 located centrally within the hydrophobic core, surrounded by aromatic and hydrophobic residues such as W467, F483, I497, and W514 (Figure 2A). Root-mean-square deviation (RMSD) analysis across the simulation period revealed enhanced flexibility in the double mutant compared to both the wild-type and single mutant forms, suggesting increased molecular mobility due to the pathogenic variants (Figure 2B). On the other hand, root-mean-square fluctuation (RMSF) analysis revealed that the loop region spanning residues 500–520 was the most flexible segment across all the mutant and wild-type protein variants (Figure 2C). The double mutant Y481H/K486N exhibited significant structural changes compared to the wild type and single mutants (Y481H and K486N). The radius of gyration (RoG) analysis showed that double mutant had a substantially higher RoG than wild type (*p* < 0.01), indicating a more expanded and less compact protein structure (Figure 2D). This increase was greater than the observed in the single mutants and was comparable to the positive pathogenic controls R482W and R482Q, previously associated with lipodystrophy. At the protein level, solvent-accessible surface area (SASA) measurements revealed that the double mutant had a significantly higher SASA than the wild-type (*p* < 0.01), suggesting increased solvent exposure and potential destabilization. This effect was more pronounced than in the single mutants and aligned closely with the positive controls. We performed residue-level SASA analysis focusing on residues 481, 482, and 486. At residue 481, the double mutant showed a significant increase in SASA compared to both wild-type and the Y481H single mutant, indicating compounded destabilization at the mutation site. Residue 482, although preserved in the Y481H/K486N double mutant, displayed increased solvent exposure, suggesting an indirect structural perturbation due to the double mutation. At residue 486, the Y481H/K486N double mutant showed a higher SASA than both the wild-type and the K486N single mutant, highlighting enhanced destabilization at this site (Figure 2E).

## Discussion

Herein, we report two concurrent *de novo* missense pathogenic variants within the *LMNA* gene (p.Y481H and p.K486N) in a patient with partial lipodystrophy. These represent a novel and intriguing genetic alteration that underscores the genetic complexity and diversity contributing to laminopathies. To the best of our knowledge, this is the first case of partial lipodystrophy syndromes of genetic origin caused by multiple simultaneous *de novo* pathogenic variants within the same gene. The pathogenicity of p.Y481H and p.K486N variants in the *LMNA* gene found in our study is supported by previous functional analysis (Vasandani et al., 2022) and our molecular modeling and DNA evolutionary analysis. It has been proposed that *LMNA* pathogenic variants cause partial lipodystrophy phenotypes via a) disruption of the structural integrity of the nuclear lamina, with special intensity in tissues exposed to frequent mechanical forces (such as muscle of limbs), and b) distortion of gene expression and chromatin organization, leading to multisystem damage, including FPLD phenotype (Xiao et al., 2023).

From a clinical perspective, our patient showed marked limb lipoatrophy with preserved adipose tissue in the face, neck, and trunk. Metabolic abnormalities included severe insulin resistance, hypertriglyceridemia, low HDL cholesterol, and fatty liver. DXA analysis revealed A/G, trunk/limb, and trunk/leg ratios indicating fat accumulation in the trunk relative to the limbs and a tendency toward central adiposity, all of which features of Dunnigan-type lipodystrophy. In contrast to patients with CGL, our patient had no glycemic disorders or hyperphagia, likely because of her remaining circulating leptin levels. Leptin deficiency seems to be the single most important determinant of the severity of metabolic abnormalities in patients with lipodystrophy and the main predictor of the clinical response to metreleptin therapy (Oral et al., 2019). *LMNA* p.R482X variants are the commonest cause of FPLD2 (Akinci et al., 2019), and their typical presentation includes limb lipoatrophy combined with preserved or excessive adiposity in the face, neck, interscapular space, intra-abdominal, and vulva. Patients with less frequent *LMNA* variants (p.R644C, p.R582H, p.R582C, p.T528M, p.N466D, p.D47N, p.R471G) are referred to as having an “atypical” FPLD and show a milder phenotype (Fernandez-Pombo et al., 2023a; Araújo-Vilar et al., 2021). Although we did not find reports on the clinical presentation of patients with FPLD2 due to the *LMNA* variants Y481H and K486N, it resembles classical FPLD2 due to p.R482 variants. This phenotypical similarity is in line with the structural consequences that we found in the molecular modeling between the double mutant *LMNA* Y481H/K486N and classical R482Q and R482W variants.

Although FPLD is typically attributed to single causative mutations, combined alterations such as those found in patients with compound heterozygous mutations in the *LMNA* gene, underscore the complex genotype/phenotype relationships in laminopathies. For example, patients with FPLD that simultaneously carries LMNA p.S583L and p.T528M mutations showed a much more severe lipoatrophy than those harboring LMNA p.S583L only, while carriers of p.T528M variant do show not lipodystrophy at all (Savage et al., 2004).

Missense gene variants in the lamin A/C C-terminal end as well as pathogenic variants in the Ig domain (amino acid positions 430–545) that prevent appropriate protein folding and stability have been associated with muscular dystrophies and lipodystrophies (Dhe-Paganon et al., 2002; Krimm et al., 2002; Östlund et al., 2001; Scharner et al., 2014). *LMNA* p.Y481H was first found in a Japanese patient with juvenile-onset proximal muscle weakness who belonged to a large family with multiple relatives affected by cardiac arrhythmias, suggesting an autosomal dominant pattern of inheritance (Kitaguchi et al., 2001). This disease was initially termed limb-girdle muscular dystrophy type 1B (LGMD1B) and subsequently renamed as Emery-Dreifuss muscular dystrophy 2 (EDMD2) (Straub et al., 2018). Interestingly, p.R482Q was reported in Emery-Dreifuss muscular dystrophy type 3 (OMIM: #616516) (Wiltshire et al., 2013), and in five Canadian probands affected with FPLD2 (OMIM: # 151660) (Cao and Hegele, 2000). Inherited disease-causing pathogenic variants in heterozygosity in the *LMNA* gene were detected for FPLD2 by positional cloning and identified in a tight cluster in different families as p.R482W, p.R482Q, p.R482L and p.K486N18. However, all the pathogenic variants reported until now were found individually across different families. In contrast, the case reported herein shows two simultaneous *de novo* mutations in the *LMNA* gene in cis (p.Y481H and p.K486N). The concurrence of these double *de novo* pathogenic variants represents a new genetic finding in FPLD2.

Both p.Y481H and p.K486N were previously evaluated through *in vitro* functional assays. The functional effect of p.Y481H was also assessed in HEK293 cells, which showed misfolding of lamin A with no protein aggregation (Anderson et al., 2021). *In vitro* analysis of p.K486N showed reduced interaction between the lamin A tail and its major posttranslational modifier ubiquitin-like modifier-1 (encoded by *SUMO1* gene) in Cos-7 cells (Anderson et al., 2021; Simon et al., 2013). Our dynamic simulation analyses suggest that p.Y481H and p.K486N, individually or combined, decrease the structural stability of the lamin A/C Ig-like domain, supporting the hypothesis that such variants compromise lamin A/C function and possibly the structural integrity of the nuclear lamina. Our findings suggest that the double mutation Y481H/K486N induces significant structural alterations in the protein, exceeding the effects of isolated single mutations. Increased RoG indicates a less compact and more expanded protein conformation, while elevated SASA at both protein and residue levels suggests enhanced solvent exposure and potential destabilization of the protein structure. The similarity of these structural changes to those observed in the pathogenic controls R482W and R482Q, also associated with FPLD, suggests that the double mutant Y481H/K486N reported herein has pathogenic implications.

The 500–520 loop plays a crucial role in maintaining the structural integrity and functional capacity of the Ig-like fold within the tail domain of lamin A/C. This region supports key interactions necessary for lamin polymerization and its association with nuclear components, including chromatin (Magracheva et al., 2009). Specifically, Pro508 is part of a groove essential for disulfide bond involving Cys522, which is critical for lamin A/C dimerization, which is necessary for the structural integrity of lamin A/C and its interaction with nuclear components (Ahn et al., 2022). In addition to its structural role, the 500–520 loop contributes indirectly to DNA binding and chromatin interactions by stabilizing the protein’s architecture. This domain has been shown to participate in DNA binding, and residues such as Lys515, Thr519, and Gln517 are key for interacting with DNA (Stierlé et al., 2003; Khromova et al., 2019). Therefore, disruption or mutations in this loop can compromise these interactions, leading to structural defects in the nuclear lamina and the onset of laminopathies, such as FPLD. Our results suggest that the Y481H and K486N mutations synergistically exacerbate structural destabilization, which could contribute to disease phenotypes (Krimm et al., 2002; Magracheva et al., 2009; Bidault et al., 2013). While bioinformatic functional prediction algorithms also support this conclusion, further functional studies are warranted to explore the biological implications of these structural changes.

*De novo* somatic or germline pathogenic variants are a wide category of genetic variations distinct from inherited variants occurring during gametogenesis and post-zygotically (Acuna-Hidalgo et al., 2016; Mohiuddin et al., 2022). Approximately 6.5% of *de novo* variants are not germline, while mosaics may occur post-zygotically derived from low-level mosaicism already present in parents (Acuna-Hidalgo et al., 2015). Our analysis suggests that both p.Y481H and p.K486N in the *LMNA* gene are germline *de novo* pathogenic variants with no mosaicism. The estimated single-nucleotide variant (SNV) mutation rate in humans leading to *de novo* variants is estimated to be ∼1.2 × 10^−8^ mutations per position per haploid genome, yielding approximately 70 new mutations in the diploid human genome, with only 1 or 2 mutations occurring within the exome (Besenbacher et al., 2016). On a megabase scale, germline mutation rates are determined by the parental age at conception, sequence context, methylation status, accessibility of the global DNA repair machinery, compaction of heterochromatin, and replication timing (Besenbacher et al., 2016; Gonzalez-Perez et al., 2019; Ségurel et al., 2014). Interestingly, late-replicating *loci* are typically clustered in heterochromatin and localize to the nuclear periphery, accumulating more germline and somatic mutations compared to early-replication *loci*, which are located near the nucleus center (Gonzalez-Perez et al., 2019; Du et al., 2019). Additional factors defining chromatin features (nucleosomes, transcription factors, and gene structure) are also important determinants of *de novo* germline mutations (Gonzalez-Perez et al., 2019). In this evolutionary context, hypermutability events within a gene may represent a mechanism with the potential to generate multiple pathogenic gene variants (Crow, 1997). Multiple closely spaced inherited and *de novo* pathogenic variants separated by <100 nucleotides have been reported (Chen et al., 2009) and referred as “mutational clusters” as opposed to multiple mutational events occurring in the same genome location in different individuals (referred to as “mutational hotspots”) (Acuna-Hidalgo et al., 2016).

Although single *de novo* pathogenic variants have been previously reported in two patients with a neonatal-onset type of lipodystrophy (Garg, 2011), the case described herein is appealing from a genetic standpoint because it occurs with two *de novo* pathogenic variants separated by only 17 nucleotides within exon 8 of the *LMNA* gene (5 amino acid residues in the lamin A/C protein). The generation of *de novo* variants may occur either as independent events or in clusters close to each other more frequently than expected by chance. Approximately 3% of human *de novo* single nucleotide variants are part of multinucleotide regions spanning a median distance of 525 bp (Besenbacher et al., 2016). A case of Ullrich congenital muscular dystrophy caused by two *de novo* pathogenic variants *in cis* (27 nucleotides apart) within the *COL6A3* gene was recently reported (Shimomura et al., 2019). *De novo* pathogenic variants can be involved in multiple diseases and are, on average, more deleterious than inherited genetic variations (Veltman and Brunner, 2012). Interestingly, multiple *de novo* missense mutations have also been reported in oncogenes of cancer patients’ tumors (Saito et al., 2021).

In summary, herein we describe the clinical and genetic characteristics of a patient with partial lipodystrophy due to two simultaneous *de novo* missense pathogenic variants *in cis* located in exon 8 of the *LMNA* gene: p.Y481H and p.K486N. Both variants were previously but independently and separately reported in muscle dystrophies and partial lipodystrophies and were causally related to lamin A/C dysfunction through *in vitro* studies. Molecular dynamics simulations provide additional insights into how these specific pathogenic variants may lead to structural instability of the lamin A/C Ig-like domain. Importantly, the clinical presentation of our patient does not correspond to the classic form of acquired partial lipodystrophy (Barraquer-Simmonds syndrome), whose etiopathogenesis remains unknown; and thus we propose to refer to this case as sporadic partial lipodystrophy of genetic origin. This sporadic form of disease caused by multiple *de novo* pathogenic variants expands the genetic and phenotypic spectrum of partial lipodystrophy syndromes and laminopathies.

## Data Availability

The data presented in this study are deposited in the UC Research Data Repository https://doi.org/10.60525/04teye511/XGXOW5.

## References

[B1] Acuna-Hidalgo R. Bo T. Kwint M. P. van de Vorst M. Pinelli M. Veltman J. A. (2015). Post-zygotic point mutations are an underrecognized source of *de novo* genomic variation. Am. J. Hum. Genet. 97 (1), 67–74. 10.1016/j.ajhg.2015.05.008 26054435 PMC4571017

[B2] Acuna-Hidalgo R. Veltman J. A. Hoischen A. (2016). New insights into the generation and role of *de novo* mutations in health and disease. Genome Biol. 17 (1), 241. 10.1186/s13059-016-1110-1 27894357 PMC5125044

[B3] Ahn J. Lee J. Jeong S. Jo I. Kang S. M. Park B. J. (2022). Structural basis for the interaction between unfarnesylated progerin and the Ig-like domain of lamin A/C in premature aging disorders. Biochem. Biophysical Res. Commun. 637, 210–217. 10.1016/j.bbrc.2022.10.070 36403485

[B4] Akinci B. Meral R. Oral E. A. (2018). Phenotypic and genetic characteristics of lipodystrophy: pathophysiology, metabolic abnormalities, and comorbidities. Curr. Diabetes Rep. 18 (12), 143. 10.1007/s11892-018-1099-9 30406415

[B5] Akinci B. Oral E. A. Neidert A. Rus D. Cheng W. Y. Thompson-Leduc P. (2019). Comorbidities and survival in patients with lipodystrophy: an international chart review study. J. Clin. Endocrinol. Metab. 104 (11), 5120–5135. 10.1210/jc.2018-02730 31314093 PMC6760298

[B6] Anderson C. L. Langer E. R. Routes T. C. McWilliams S. F. Bereslavskyy I. Kamp T. J. (2021). Most myopathic lamin variants aggregate: a functional genomics approach for assessing variants of uncertain significance. npj Genomic Med. 6 (1), 103. 10.1038/s41525-021-00265-x PMC8642518 34862408

[B7] Araújo-Vilar D. Sánchez-Iglesias S. Castro A. I. Cobelo-Gómez S. Hermida-Ameijeiras Á. Rodríguez-Carnero G. (2021). Variable expressivity in type 2 familial partial lipodystrophy related to R482 and N466 variants in the LMNA gene. J. Clin. Med. 10 (6), 1259. 10.3390/jcm10061259 33803652 PMC8002937

[B8] Besenbacher S. Sulem P. Helgason A. Helgason H. Kristjansson H. Jonasdottir A. (2016). Multi-nucleotide *de novo* mutations in humans. PLOS Genet. 12 (11), e1006315. 10.1371/journal.pgen.1006315 27846220 PMC5147774

[B9] Bidault G. Garcia M. Vantyghem M. C. Ducluzeau P. H. Morichon R. Thiyagarajah K. (2013). Lipodystrophy-linked LMNA p.R482W mutation induces clinical early atherosclerosis and *in vitro* endothelial dysfunction. Arterioscler. Thromb. Vasc. Biol. 33 (9), 2162–2171. 10.1161/ATVBAHA.113.301933 23846499

[B10] Brown R. J. Araujo-Vilar D. Cheung P. T. Dunger D. Garg A. Jack M. (2016). The diagnosis and management of lipodystrophy syndromes: a multi-society practice guideline. J. Clin. Endocrinol. and Metabolism 101 (12), 4500–4511. 10.1210/jc.2016-2466 PMC5155679 27710244

[B11] Cao H. Hegele R. A. (2000). Nuclear lamin A/C R482Q mutation in Canadian kindreds with Dunnigan-type familial partial lipodystrophy. Hum. Mol. Genet. 9 (1), 109–112. 10.1093/hmg/9.1.109 10587585

[B12] Casasola A. Scalzo D. Nandakumar V. Halow J. Recillas-Targa F. Groudine M. (2016). Prelamin A processing, accumulation and distribution in normal cells and laminopathy disorders. Nucleus 7 (1), 84–102. 10.1080/19491034.2016.1150397 26900797 PMC4916894

[B13] Chen J.-M. Férec C. Cooper D. N. (2009). Closely spaced multiple mutations as potential signatures of transient hypermutability in human genes. Hum. Mutat. 30 (10), 1435–1448. 10.1002/humu.21088 19685533

[B14] Cortés V. Santos J. L. (2019). Clinical presentation and treatment of primary lipodystrophies. Rev. Med. Chil. 147 (11), 1449–1457. 10.4067/S0034-98872019001101449 32186606

[B15] Cortés V. A. Curtis D. E. Sukumaran S. Shao X. Parameswara V. Rashid S. (2009). Molecular mechanisms of hepatic steatosis and insulin resistance in the AGPAT2-deficient mouse model of congenital generalized lipodystrophy. Cell Metab. 9 (2), 165–176. 10.1016/j.cmet.2009.01.002 19187773 PMC2673980

[B16] Cortés V. A. Smalley S. V. Goldenberg D. Lagos C. F. Hodgson M. I. Santos J. L. (2014). Divergent metabolic phenotype between two sisters with congenital generalized lipodystrophy due to double AGPAT2 homozygous mutations. A clinical, genetic and *in silico* study. PLOS ONE 9 (1), e87173. 10.1371/journal.pone.0087173 24498038 PMC3909042

[B17] Crasto S. My I. Di Pasquale E. (2020). The broad spectrum of LMNA cardiac diseases: from molecular mechanisms to clinical phenotype. Front. Physiology 11, 761. 10.3389/fphys.2020.00761 PMC7349320 32719615

[B18] Crow J. F. (1997). The high spontaneous mutation rate: is it a health risk? Proc. Natl. Acad. Sci. U. S. A. 94 (16), 8380–8386. 10.1073/pnas.94.16.8380 9237985 PMC33757

[B19] Dhe-Paganon S. Werner E. D. Chi Y.-I. Shoelson S. E. (2002). Structure of the globular tail of nuclear lamin. J. Biol. Chem. 277 (20), 17381–17384. 10.1074/jbc.C200038200 11901143

[B20] Dittmer T. A. Misteli T. (2011). The lamin protein family. Genome Biol. 12 (5), 222. 10.1186/gb-2011-12-5-222 21639948 PMC3219962

[B21] Du Q. Bert S. A. Armstrong N. J. Caldon C. E. Song J. Z. Nair S. S. (2019). Replication timing and epigenome remodelling are associated with the nature of chromosomal rearrangements in cancer. Nat. Commun. 10 (1), 416. 10.1038/s41467-019-08302-1 30679435 PMC6345877

[B22] Fernandez-Pombo A. Diaz-Lopez E. J. Castro A. I. Sanchez-Iglesias S. Cobelo-Gomez S. Prado-Moraña T. (2023a). Clinical spectrum of LMNA-associated type 2 familial partial lipodystrophy: a systematic review. Cells 12 (5), 725. 10.3390/cells12050725 36899861 PMC10000975

[B23] Fernandez-Pombo A. Sánchez-Iglesias S. Castro-Pais A. I. Ginzo-Villamayor M. J. Cobelo-Gómez S. Prado-Moraña T. (2023b). Natural history and comorbidities of generalised and partial lipodystrophy syndromes in Spain. Front. Endocrinol. (Lausanne) 14, 1250203. 10.3389/fendo.2023.1250203 38034001 PMC10687442

[B24] Florwick A. Dharmaraj T. Jurgens J. Valle D. Wilson K. L. (2017). LMNA sequences of 60,706 unrelated individuals reveal 132 novel missense variants in A-type lamins and suggest a link between variant p.G602S and type 2 diabetes. Front. Genet. 8, 79. 10.3389/fgene.2017.00079 28663758 PMC5471320

[B25] Frazer J. Notin P. Dias M. Gomez A. Min J. K. Brock K. (2021). Disease variant prediction with deep generative models of evolutionary data. Nature 599 (7883), 91–95. 10.1038/s41586-021-04043-8 34707284

[B26] Garg A. (2011). Clinical review#: lipodystrophies: genetic and acquired body fat disorders. J. Clin. Endocrinol. and Metabolism 96 (11), 3313–3325. 10.1210/jc.2011-1159 PMC7673254 21865368

[B27] Genschel J. Schmidt H. H. J. (2000). Mutations in the LMNA gene encoding lamin A/C. Hum. Mutat. 16 (6), 451–459. 10.1002/1098-1004(200012)16:6<451::AID-HUMU1>3.0.CO;2-9 11102973

[B28] Gonzalez-Perez A. Sabarinathan R. Lopez-Bigas N. (2019). Local determinants of the mutational landscape of the human genome. Cell 177 (1), 101–114. 10.1016/j.cell.2019.02.051 30901533

[B29] Huang Y. Xiao Y. Xue J. Zhang L. Wang L. (2024). Development of a coding SNP panel for tracking the origin of whole-exome sequencing samples. BMC Genomics 25 (1), 142. 10.1186/s12864-024-10052-4 38317084 PMC10840194

[B30] Imboden M. T. Welch W. A. Swartz A. M. Montoye A. H. K. Finch H. W. Harber M. P. (2017). Reference standards for body fat measures using GE dual energy x-ray absorptiometry in Caucasian adults. PLoS One 12 (4), e0175110. 10.1371/journal.pone.0175110 28388669 PMC5384668

[B31] Khromova N. V. Perepelina K. I. Ivanova O. A. Malashicheva A. B. Kostareva A. A. Dmitrieva R. I. (2019). R482L mutation of the LMNA gene affects dynamics of C2C12 myogenic differentiation and stimulates formation of intramuscular lipid droplets. Biochem. Mosc. 84 (3), 241–249. 10.1134/S0006297919030064 31221062

[B32] Kitaguchi T. Matsubara S. Sato M. Miyamoto K. Hirai S. Schwartz K. (2001). A missense mutation in the exon 8 of lamin A/C gene in a Japanese case of autosomal dominant limb-girdle muscular dystrophy and cardiac conduction block. Neuromuscul. Disord. 11 (6), 542–546. 10.1016/s0960-8966(01)00207-3 11525883

[B33] Krimm I. Ostlund C. Gilquin B. Couprie J. Hossenlopp P. Mornon J. P. (2002). The ig-like structure of the C-terminal domain of lamin A/C, mutated in muscular dystrophies, cardiomyopathy, and partial lipodystrophy. Structure 10 (6), 811–823. 10.1016/s0969-2126(02)00777-3 12057196

[B34] Lindquist P. Gasbjerg L. S. Mokrosinski J. Holst J. J. Hauser A. S. Rosenkilde M. M. (2022). The location of missense variants in the human GIP gene is indicative for natural selection. Front. Endocrinol. (Lausanne) 13, 891586. 10.3389/fendo.2022.891586 35846282 PMC9277503

[B35] Magracheva E. Kozlov S. Stewart C. L. Wlodawer A. Zdanov A. (2009). Structure of the lamin A/C R482W mutant responsible for dominant familial partial lipodystrophy (FPLD). Acta Crystallogr. Sect. F. Struct. Biol. Cryst. Commun. 65 (Pt 7), 665–670. 10.1107/S1744309109020302 PMC2705630 19574635

[B36] Miranda J. P. Pereira A. Corvalán C. Miquel J. F. Alberti G. Gana J. C. (2024). Genetic determinants of serum bilirubin using inferred native American gene variants in Chilean adolescents. Front. Genet. 15, 1382103. 10.3389/fgene.2024.1382103 38826804 PMC11140026

[B37] Mohiuddin M. Kooy R. F. Pearson C. E. (2022). *De novo* mutations, genetic mosaicism and human disease. Front. Genet. 13, 983668. 10.3389/fgene.2022.983668 36226191 PMC9550265

[B38] Monteiro L. Foss-Freitas M. C. Navarro A. Pereira F. Coeli F. Carneseca E. (2017). Evaluation of dietary intake, leisure-time physical activity, and metabolic profile in women with mutation in the LMNA gene. J. Am. Coll. Nutr. 36 (4), 248–252. 10.1080/07315724.2016.1262299 28443701

[B39] Nishiuchi S. Makiyama T. Aiba T. Nakajima K. Hirose S. Kohjitani H. (2017). Gene-based risk stratification for cardiac disorders in LMNA mutation carriers. Circ. Cardiovasc. Genet. 10 (6), e001603. 10.1161/CIRCGENETICS.116.001603 29237675

[B40] Oral E. A. Gorden P. Cochran E. Araújo-Vilar D. Savage D. B. (2019). Long-term effectiveness and safety of metreleptin in the treatment of patients with partial lipodystrophy. Endocrine 64 (3), 500–511. 10.1007/s12020-019-01862-8 30805888 PMC7340120

[B41] Östlund C. Bonne G. l. Schwartz K. Worman H. J. (2001). Properties of lamin A mutants found in Emery-Dreifuss muscular dystrophy, cardiomyopathy and Dunnigan-type partial lipodystrophy. J. Cell Sci. 114 (24), 4435–4445. 10.1242/jcs.114.24.4435 11792809

[B42] Richards S. Aziz N. Bale S. Bick D. Das S. Gastier-Foster J. (2015). Standards and guidelines for the interpretation of sequence variants: a joint consensus recommendation of the American college of medical genetics and genomics and the association for molecular pathology. Genet. Med. 17 (5), 405–424. 10.1038/gim.2015.30 25741868 PMC4544753

[B43] Saito Y. Koya J. Kataoka K. (2021). Multiple mutations within individual oncogenes. Cancer Sci. 112 (2), 483–489. 10.1111/cas.14699 33073435 PMC7894016

[B44] Santos J. L. Cortés V. A. (2021). Eating behaviour in contrasting adiposity phenotypes: monogenic obesity and congenital generalized lipodystrophy. Obes. Rev. 22 (1), e13114. 10.1111/obr.13114 33030294

[B45] Savage D. B. Soos M. A. Powlson A. O'Rahilly S. McFarlane I. Halsall D. J. (2004). Familial partial lipodystrophy associated with compound heterozygosity for novel mutations in the LMNA gene. Diabetologia 47 (4), 753–756. 10.1007/s00125-004-1360-4 15298354

[B46] Scharner J. Lu H. C. Fraternali F. Ellis J. A. Zammit P. S. (2014). Mapping disease-related missense mutations in the immunoglobulin-like fold domain of lamin A/C reveals novel genotype–phenotype associations for laminopathies. Proteins Struct. Funct. Bioinforma. 82 (6), 904–915. 10.1002/prot.24465 24375749

[B47] Ségurel L. Wyman M. J. Przeworski M. (2014). Determinants of mutation rate variation in the human germline. Annu. Rev. Genomics Hum. Genet. 15 (1), 47–70. 10.1146/annurev-genom-031714-125740 25000986

[B48] Shackleton S. Lloyd D. J. Jackson S. N. Evans R. Niermeijer M. F. Singh B. M. (2000). LMNA, encoding lamin A/C, is mutated in partial lipodystrophy. Nat. Genet. 24 (2), 153–156. 10.1038/72807 10655060

[B49] Shimomura H. Lee T. Tanaka Y. Awano H. Itoh K. Nishino I. (2019). Two closely spaced mutations in cis result in Ullrich congenital muscular dystrophy. Hum. Genome Var. 6 (1), 21. 10.1038/s41439-019-0052-z 31044083 PMC6486579

[B50] Simon D. N. Domaradzki T. Hofmann W. A. Wilson K. L. (2013). Lamin A tail modification by SUMO1 is disrupted by familial partial lipodystrophy-causing mutations. Mol. Biol. Cell 24 (3), 342–350. 10.1091/mbc.E12-07-0527 23243001 PMC3564541

[B51] Stierlé V. Couprie J. Ostlund C. Krimm I. Zinn-Justin S. Hossenlopp P. (2003). The carboxyl-terminal region common to lamins A and C contains a DNA binding domain. Biochemistry 42 (17), 4819–4828. 10.1021/bi020704g 12718522

[B52] Straub V. Murphy A. Udd B. LGMD workshop study group (2018). 229th ENMC international workshop: limb girdle muscular dystrophies – nomenclature and reformed classification Naarden, The Netherlands, 17–19 March 2017. Neuromuscul. Disord. 28 (8), 702–710. 10.1016/j.nmd.2018.05.007 30055862

[B53] Vasandani C. Li X. Sekizkardes H. Brown R. J. Garg A. (2022). Phenotypic differences among familial partial lipodystrophy due to LMNA or PPARG variants. J. Endocr. Soc. 6 (12), bvac155. 10.1210/jendso/bvac155 36397776 PMC9664976

[B54] Veltman J. A. Brunner H. G. (2012). *De novo* mutations in human genetic disease. Nat. Rev. Genet. 13 (8), 565–575. 10.1038/nrg3241 22805709

[B55] Wiltshire K. M. Hegele R. A. Innes A. M. Brownell A. K. (2013). Homozygous lamin A/C familial lipodystrophy R482Q mutation in autosomal recessive Emery Dreifuss muscular dystrophy. Neuromuscul. Disord. 23 (3), 265–268. 10.1016/j.nmd.2012.11.011 23313286

[B56] Xiao C. Liu J. Yang C. Zhai X. Liu P. Xiao X. (2023). The clinical characteristics and potential molecular mechanism of LMNA mutation-related lipodystrophy. Adv. Biol. n/a (n/a), 2200301. 10.1002/adbi.202200301 37303127

