# Supplementary material for: Case Report: Concurrent de novo pathogenic variants in the LMNA gene as a cause of sporadic partial lipodystrophy
Source: Front Genet. 2024 Nov 28;15:1468878. doi: 10.3389/fgene.2024.1468878 (PMC11634843; doi:10.3389/fgene.2024.1468878)
Supplement: Supplementary file 1 [file DataSheet1.pdf]

Supplementary Material of

Concurrent *de novo* pathogenic variants in the *LMNA* gene as a cause of sporadic partial lipodystrophy

Supplementary Data

Methods for molecular modeling and dynamic simulations of Lamin A/C variants

The first conformer of the NMR ensemble of human lamin A/C (residues 429-549, PDB id 1IVT) was used to generate variants Y481H, K486N, Y481H:K486N double mutant (DoubMut), and R482W and R482Q control pathogenic variants (Krimm et al., 2002). We used molecular dynamics simulations to investigate the structural repercussions of such variants in the Ig-like domain of lamin A/C, which have been implicated in familial partial lipodystrophy and muscle dystrophy disorders. Wild-type (WT) and lamin A/C pathogenic variants were solvated and ionized using the CHARMM-GUI web server interface (Jo et al., 2008). The proteins were embedded in a water box of 65x65x65 Å, and the system charges were equilibrated with Na+ and Cl- atoms up to a concentration of 0.15 M NaCl. The final systems contained approximately 26,700 atoms. All molecular dynamics simulations were performed using the NAMD v2.14 package (Phillips et al., 2020).The simulation systems were first relaxed with 10,000 steps of minimization followed by gradual heating from 0 to 310 K by running short molecular dynamics simulations of 500 steps each cycle using the canonical NVT ensemble (N: number of particles in the system; V: volume; T: temperature). The simulation was switched to constant NPT conditions (constant temperature; constant pressure) and further equilibrated for 2.5 ns while constraining the protein backbone with an initial force constant of 10 kcal/(mol·Å2) and gradually decreased to 8, 6, 4, 2, 1, 0.5, and 0.05 kcal/(mol·Å2) every 250 ps of molecular dynamics simulation. Finally, the systems were run without any constraints for 100 ns. The protein, cofactor, and ion atom types and parameters used were described by the CHARMM36 force field (Huang and MacKerell Jr, 2013). Van der Waals interactions were calculated by applying a cutoff distance of 12 Å and switching the potential from 10 Å. A timestep of 2 fs was used in the production phase, while PME (Particle Mesh Ewald) was employed for the treatment of long-range electrostatic interactions. The temperature was maintained at 310°K using Langevin dynamics. The Nose–Hoover–Langevin piston method was used to control the target pressure (1 atm), with the Langevin Piston Period set to 200 fs and the Langevin Piston Decay set to 50 fs. Trajectory analysis was performed using Visual Molecular Dynamics v1.94 (VMD) software (Humphrey et al., 1996). The effect of the gene variants on the structure was first explored by measuring the root-mean-square deviation of carbon atoms (RMSD) and the root-mean square fluctuation of individual residues (RMSF) against the first frame of the production phase. Then, the frames corresponding to the last 50 ns (500 frames) of each simulation was extracted to compare the radius of gyration (RoG), and the solvent-accessible surface area (SASA) for the proteins and individual residues through equilibrium simulation. One-way ANOVA followed by Bonferroni's multiple comparisons test was performed using GraphPad Prism version 10.3.1 (GraphPad Software Inc). A p-value less than 0.05 was considered to indicate statistical significance.

References for the molecular modeling section

Huang, J., and MacKerell Jr, A.D. (2013). CHARMM36 all-atom additive protein force field: Validation based on comparison to NMR data. *Journal of Computational Chemistry* 34(25)**,** 2135-2145. doi: https://doi.org/10.1002/jcc.23354.

Humphrey, W., Dalke, A., and Schulten, K. (1996). VMD: Visual molecular dynamics. *Journal of Molecular Graphics* 14(1)**,** 33-38. doi: https://doi.org/10.1016/0263-7855(96)00018-5.

Jo, S., Kim, T., Iyer, V.G., and Im, W. (2008). CHARMM-GUI: A web-based graphical user interface for CHARMM. *Journal of Computational Chemistry* 29(11)**,** 1859-1865. doi: https://doi.org/10.1002/jcc.20945.

Krimm, I., Östlund, C., Gilquin, B., Couprie, J., Hossenlopp, P., Mornon, J.-P., et al. (2002). The Ig-like Structure of the C-Terminal Domain of Lamin A/C, Mutated in Muscular Dystrophies, Cardiomyopathy, and Partial Lipodystrophy. *Structure* 10(6)**,** 811-823. doi: https://doi.org/10.1016/S0969-2126(02)00777-3.

Phillips, J.C., Hardy, D.J., Maia, J.D.C., Stone, J.E., Ribeiro, J.V., Bernardi, R.C., et al. (2020). Scalable molecular dynamics on CPU and GPU architectures with NAMD. *The Journal of Chemical Physics* 153(4)**,** 044130. doi: 10.1063/5.0014475.

Sanger sequencing primers for the region of interest in the *LMNA* gene

The primers used for the Sanger sequencing were the following:

Primer forward F5'->3' (LMNA): CTCTTCCCTATCTTCCCGGC

Primer reverse R5'->3' (LMNA): AGGCCCAGGCCTACTTACTTC

The amplicon size was 352 bp. Capital letters in red indicate the *de novo* mutations found in the patient.

>chr1:156136899-156137250
CTCTTCCCTATCTTCCCGGCaggaccagtccatgggcaattggcagatcaagcgccagaatggagatgatcccttgctgact**T**
accggttcccaccaaa**G**ttcaccctgaaggctgggcaggtggtgacggtgagtggcagggcgcttgggactctggggaggccttgggtggcg
atgggagcgctggggtaagtgtccttttctcctctccagatctgggctgcaggagctggggccacccacagccccccctaccgacctggtgtggaa
ggcacagaacacctggggctgcgggaacagcctgcgtacggctctcatcaactccactgggGAAGTAAGTAGGCCTGGGCC
T

Figure S1. Graphical representation of the DNA and amino acid sequence conservation in the *LMNA* region containing p.Y481H (rs59981161) and p.K486N (rs57747780)

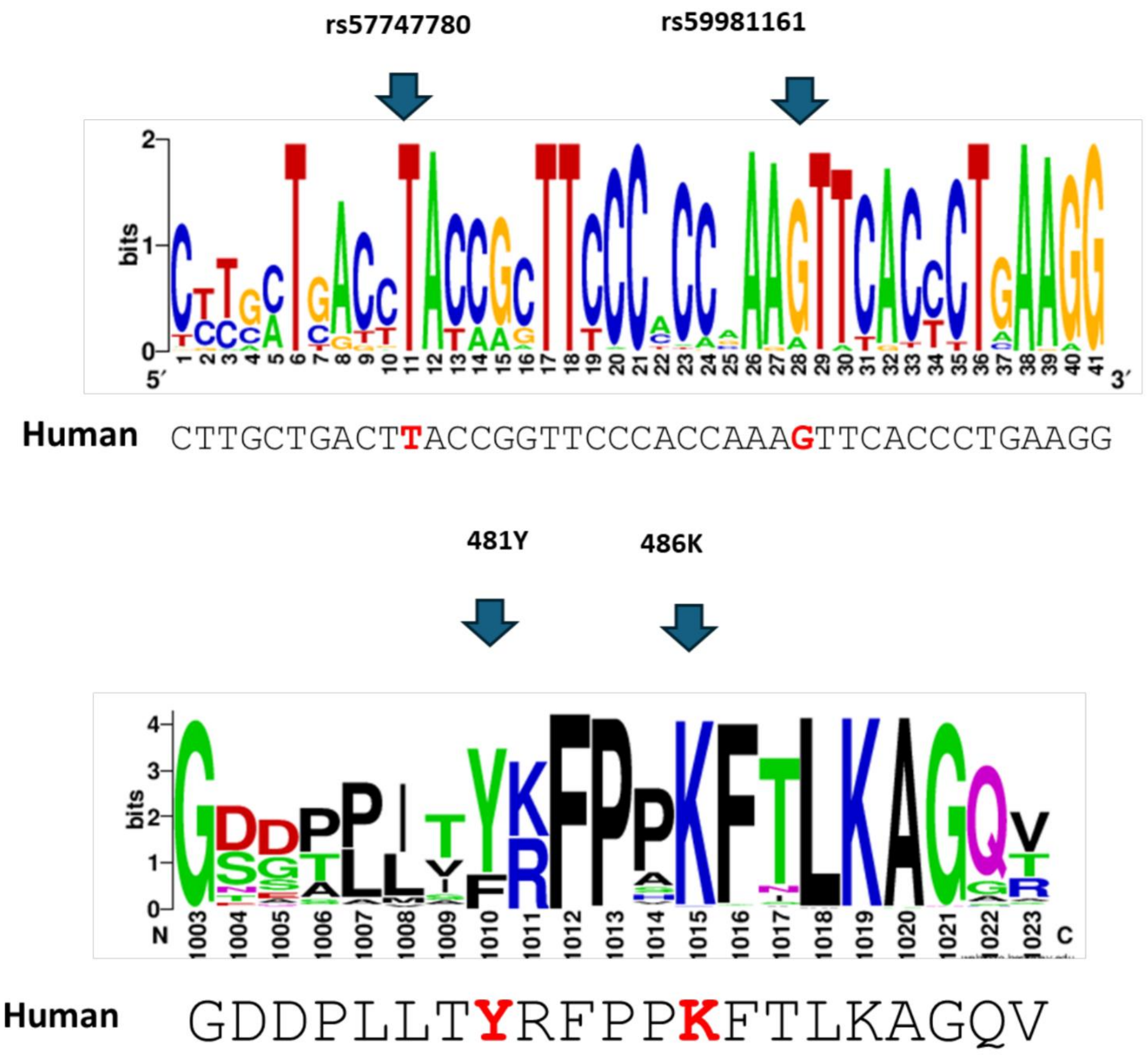

Based on orthologue alignments of 245 species retrieved from Ensembl (https://www.ensembl.org/). The logo plots were created with https://weblogo.berkeley.edu/
https://www.ensembl.org/Homo_sapiens/Gene/Compara_Ortholog?db=core;g=ENSG00000160789;r=1:156082572-156140081

Crooks GE, Hon G, Chandonia JM, Brenner SE. WebLogo: A Sequence Logo Generator. Genome Res (2004) 14(6):1188–90. doi: 10.1101/gr.849004

Figure S2. Nucleotide sequence in the hg19 human genome assembly chr1:156,106,751-156,106,810 and comparison to 46 vertebrates

Human:ggagatgatcccttgctgact**T**accggttcccaccaaa**G**ttcaccctgaaggctgggcag

The rs57747780 variant (p.Tyr481His) is in chr1: 156,106,772 (T>C) (shown above in red)
The rs59981161 variant (p.Lys486Asn) is in chr1: 156,106,789 (G>T) (shown above in red)

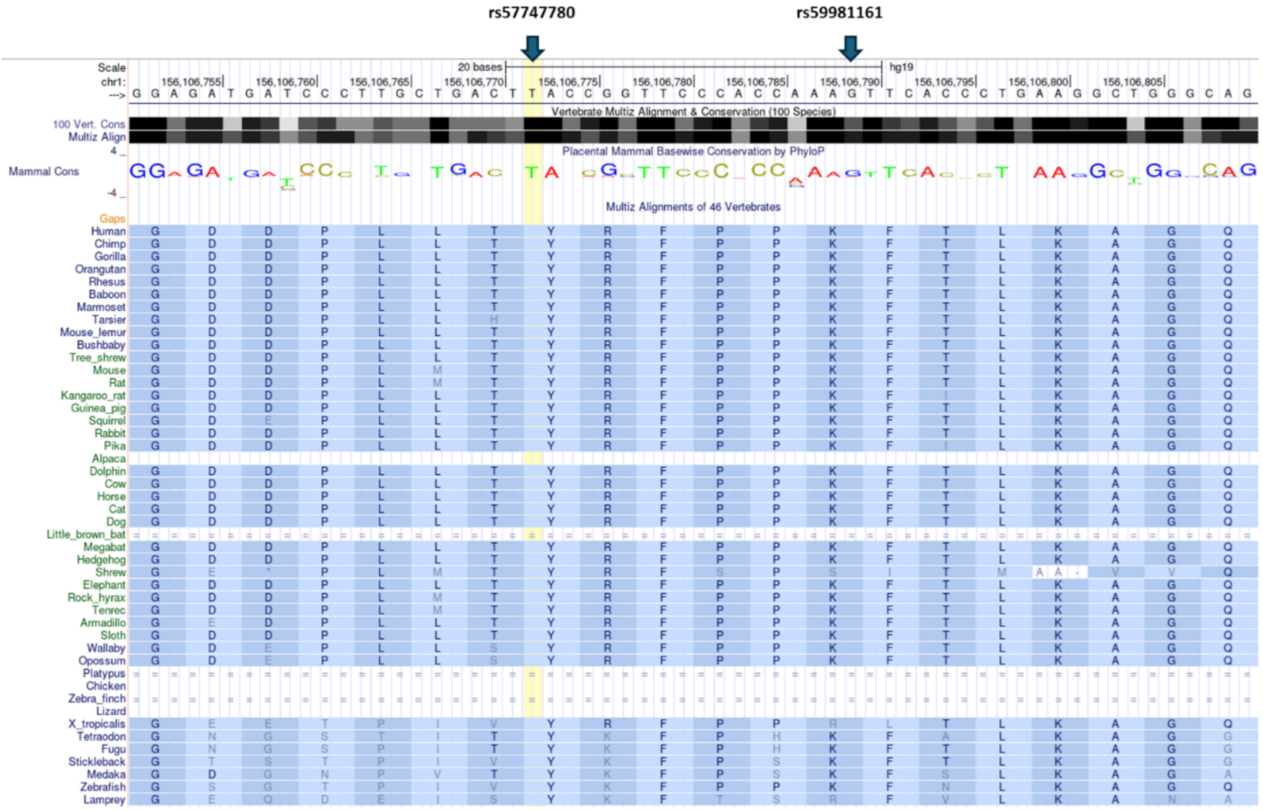

Derived from:

https://genome.ucsc.edu/cgi-bin/hgTracks?db=hg19&lastVirtModeType=default&lastVirtModeExtraState=&virtModeType=default&virtMode=0&nonVirtPosition=&position=chr1%3A156106751%2D156106810&hgsid=2346597470_RRBh3uiAzXEyY6z4mvuSLU8UIAbL

https://genome.ucsc.edu/cgi-bin/hgTrackUi?db=hg19&g=cons100way

Figure S3. Conservation scores from the CONSURF software in the human *LMNA* gene

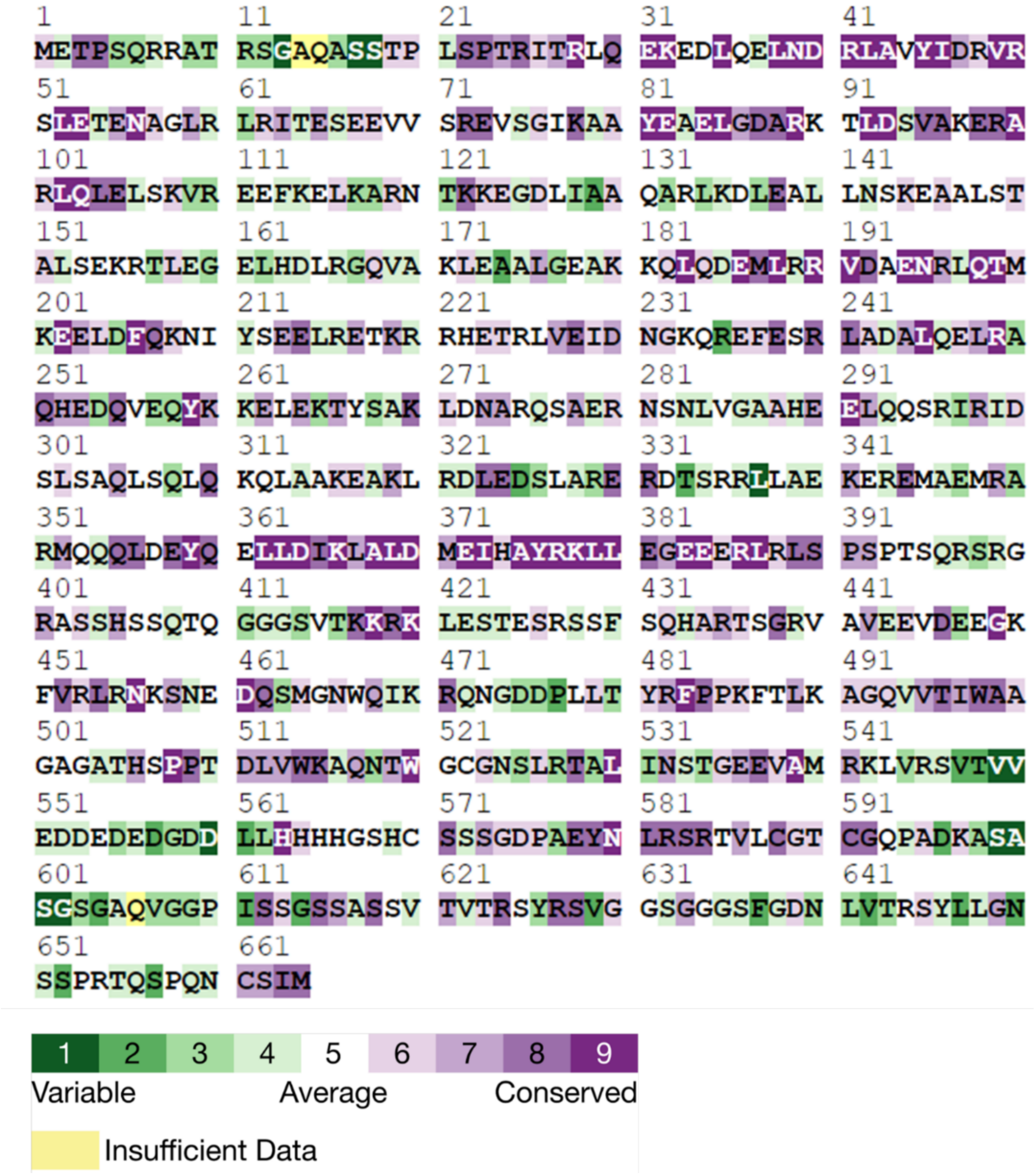

Score 481Y: -0.39 (95%CI: -0.661, -0.149). Grade: 6
Score 486K: - 0.373 (95%CI: -0.661, -0.250). Grade: 6

Derived from: https://consurf.tau.ac.il/

Ashkenazy H, Abadi S, Martz E, Chay O, Mayrose I, Pupko T, et al. ConSurf 2016: An Improved Methodology to Estimate and Visualize Evolutionary Conservation in Macromolecules. Nucleic Acids Res (2016) 44(W1):W344–50

Table S1. Features of Single Nucleotide Polymorphisms (SNPs) used for paternity assessment in the Chilean population

SNP rsID	Chrom.	Position (hg19)	In MEGA-Illumina GOCS-Chile$	Freq Ref. Allele &	Freq Alt. Allele &	P-value HWE (GOCS-Chile)	Discrimination Capacity (DC)	Probability of Exclusion (PE)
rs12221474	10	99,332,488	Imputed	0.694 (A)	0.306 (C)	0.18	0.583	0.117
rs5960	13	113,801,737	Measured	0.308 (C)	0.692 (T)	0.6	0.578	0.136
rs6061243	20	61,040,453	Imputed	0.557 (C)	0.443 (G)	0.24	0.631	0.136
rs1128925	19	2,767,192	Imputed	0.71 (G)	0.29 (T)	0.53	0.571	0.115
rs9620123	22	43,614,316	Imputed	0.83 (C)	0.17 (G)	0.81	0.449	0.057
rs8048410	16	1,614,097	Measured	0.722 (A)	0.278 (G)	0.2	0.565	0.105
rs6503070	17	7,948,175	Measured	0.408 (C)	0.592 (T)	0.63	0.612	0.180
rs231399	4	2,831,383	Measured	0.343 (T)	0.657 (G)	0.62	0.600	0.142
rs12990557	2	202,342,402	Imputed	0.503 (G)	0.497 (T)	0.61	0.629	0.180
rs2297079	9	421,032	Imputed	0.62 (C)	0.38 (G)	0.95	0.609	0.180
rs12179	7	150,557,622	Measured	0.667 (G)	0.333 (A)	0.77	0.592	0.148
rs3734557	6	40,360,465	Measured	0.622 (A)	0.378 (G)	0.76	0.611	0.159
rs1051614	1	154,744,807	Imputed	0.561 (C)	0.439 (G)	0.47	0.615	0.192
rs2279819	3	125,726,048	Imputed	0.549 (G)	0.451 (C)	0.28	0.613	0.162
rs2304035	5	168,176,517	Measured	0.44 (A)	0.56 (G)	0.47	0.627	0.172
rs6559167#	8	6,389,889	Not available	0.396 (C)	0.31 (A) /0.29 (G)	-	0.660	0.402
rs3741097	11	134,244,123	Imputed	0.809 (C)	0.191 (G)	0.46	0.476	0.060
rs4758686	12	122,623,000	Imputed	0.453 (T)	0.547 (C)	0.85	0.621	0.187
rs3737171	14	57,052,511	Measured	0.472 (G)	0.528 (T)	0.9	0.625	0.184
rs3744877	18	77,894,844	Measured	0.407 (G)	0.593 (A)	0.5	0.620	0.163
rs2249057	21	47,773,103	Imputed	0.752 (C)	0.248 (A)	0.66	0.539	0.100
rs3743399	15	89,398,330	Imputed	0.351 (G)	0.649 (A)	1.0	0.599	0.151

List of SNPs based on Huang Y, Xiao Y, Qu S, Xue J, Zhang L, Wang L, Liang W. Development of a coding SNP panel for tracking the origin of whole-exome sequencing samples. BMC Genomics 2024; 25(1):142.

$ Genotypes in the Chilean population were retrieved from the Growth and Obesity Cohort Study (GOCS) using the MEGA-Illumina array (www.illumina.com) (Miranda JP, Pereira A, Corvalán C, Miquel JF, Alberti G, Gana JC, Santos JL. Genetic determinants of serum bilirubin using inferred native American gene variants in Chilean adolescents. Front Genet 2024; 15:1382103). Imputation was carried out in the TOPMed imputation server (https://imputation.biodatacatalyst.nhlbi.nih.gov/#!).

& Allele frequencies (reference and alternative alleles) correspond to the Chilean population, and derived from the Chilean GOCS, except for rs6559167 (see below)

Discrimination capacity and probability of exclusion were calculated using FORSTAT https://fdl-uwc.shinyapps.io/forstat/, (Ristow PG, D'Amato ME. Forensic statistics analysis toolbox (FORSTAT): A streamlined workflow for forensic statistics. Forensic Science International: Genetics Supplement Series 2017; 6: e52–e54). Exact p-values for Hardy-Weinberg Equilibrium (HWE) were calculated using GENEPOP program https://genepop.curtin.edu.au/genepop_op1.html

Allele frequencies, discrimination capacity, and probability of exclusion of rs6559167 were taken from Huang et al. (2024) using data from the American population (1000 genomes)

Table S2. Paternity index (PI), combined paternity index (CPI), and probability of paternity (W) in the case-parents trio

SNP rsID	Ref. Allele	Alt. Allele	Depth of MPS exome reads: Case	Depth of MPS exome reads: Mother	Depth of MPS exome reads: Father	Genotypes: Case	Genotypes: Mother	Genotypes: Father	Paternity Index (PI)
rs12221474	A	C	A: 34 C:0	A:44 C:0	A:36 C:0	A/A	A/A	A/A	1,44
rs5960	C	T	C:102	C: 92 T:0	C:117	C/C	C/C	C/C	3,25
rs6061243	C	G	C:0 G:48	C:0 G:39	C:0 G:45	G/G	G/G	G/G	2,26
rs1128925	G	T	G:9 T:14	G:16 T:0	G:0 T:14	G/T	G/G	T/T	3,45
rs9620123	C	G	C:24	C:26	C:23	C/G	C/G	C/G	1,00
rs8048410	A	G	A:1 G:72	A:45	A:0 G:68	G/G	A/G	G/G	3,60
rs6503070	C	T	C:20	C:30	C:18	C/T	C/T	C/T	1,00
rs231399	T	G	T:0 G:99	T:1 G:96	T:0 G:71	G/G	G/G	G/G	1,52
rs12990557	G	T	G:0 T:81	G:30	G:29	T/T	G/T	G/T	1,01
rs2297079	C	G	C:23	C:0 G:41	C:18	C/G	G/G	C/G	1,32
rs12179	G	A	G:55	G:35	G:36	G/A	G/A	G/A	1,00
rs3734557	A	G	A:128	A:123	A:110	A/A	A/A	A/A	1,61
rs1051614	C	G	C:58	C:124	C:56	C/G	C/C	C/G	0,89
rs2279819	G	C	G:57 C:1	G:47 C:0	G:62 C:0	G/G	G/G	G/G	1,82
rs2304035	A	G	A:0 G:72	A:21	A:37	G/G	A/G	A/G	0,89
rs6559167	C	A/G	C:46	C:0 G:54	C:43	C/G	G/G	C/A	1,72
rs3741097	C	G	C:100	C:80 G:0	C:106	C/C	C/C	C/C	1,24
rs4758686	T	C	T:0 C:54	T:0 C:52	T:0 C:46	C/C	C/C	C/C	1,83
rs3737171	G	T	G:0 T:55	G:0 T:61	G:0 T:60	T/T	T/T	T/T	1,89
rs3744877	G	A	G:48	G:98 A:0	G:0	G/A	G/G	A/A	1,69
rs2249057	C	A	C:31 A:0	C:47 A:0	C:29 A:0	C/C	C/C	C/C	1,33
rs3743399	G	A	G:47	G:105	G:39	G/A	G/G	G/A	1,42

Combined paternity index (CPI) = 14498

Probability of Paternity (W) = 0.999931

List of SNPs based on Huang Y, Xiao Y, Qu S, Xue J, Zhang L, Wang L, Liang W. Development of a coding SNP panel for tracking the origin of whole-exome sequencing samples. BMC Genomics 2024; 25(1):142.

MPS: Massive-parallel sequencing

Calculations of parentage statistics are based on https://worldwide.promega.com/es-es/products/pm/genetic-identity/ishi-conference-proceedings/15th-ishi-parentage-and-mixture-statistics-workshop/ and in accordance with Gjertson DW, Brenner CH, Baur MP, Carracedo A, Guidet F, Luque JA, Lessig R, Mayr WR, Pascali VL, Prinz M, Schneider PM, Morling N. ISFG: Recommendations on biostatistics in paternity testing. Forensic Sci Int Genet 2007; 1: 223-31.

The probability of paternity (W) calculated using allele frequencies from the American population of 1000 genomes (instead of Chilean GOCS) was estimated as W=0.999932, with a Combined paternity index-CPI=14640.
